# Novel Role of 5-Methyl-(6S)-Tetrahydrofolate in Mediating Endothelial Cell Tetrahydrobiopterin in Pregnancy and Implications for Gestational Hypertension

**DOI:** 10.1161/HYPERTENSIONAHA.124.22838

**Published:** 2024-07-23

**Authors:** Yasmin Dickinson, Ruth Boehni, Rima Obeid, Jean-Pierre Knapp, Rudolf Moser, Adam J. Lewandowski, Gillian Douglas, Paul Leeson, Keith M. Channon, Surawee Chuaiphichai

**Affiliations:** Division of Cardiovascular Medicine, British Heart Foundation Centre of Research Excellence, Radcliffe Department of Medicine (Y.D., G.D., K.M.C., S.C.); Oxford Cardiovascular Clinical Research Facility, Division of Cardiovascular Medicine, Radcliffe Department of Medicine (P.L.); Nuffield Department of Population Health (A.J.L.); University of Oxford, United Kingdom. Merck & Cie KmG Schaffhausen, Switzerland (R.B., J.-P.K., R.M.); Department of Clinical Chemistry and Laboratory Medicine, Saarland University Hospital Homburg, Saar, Germany (R.O.).

**Keywords:** endothelial cells, folic acid, human, hypertension, mice, nitric oxide synthase, pregnancy

## Abstract

**BACKGROUND:**

Folate intake during pregnancy is essential for fetal development and maternal health. However, the specific effects of folic acid (FA) and 5-methyl-(6S)-tetrahydrofolate (5-MTHF) on the prevention and treatment of hypertensive disorders of pregnancy remain unclear. We investigated whether FA and 5-MTHF have different effects on endothelial cell tetrahydrobiopterin (BH4) metabolism in pregnancy and the possible consequences for endothelial NO generation, maternal blood pressure, and fetal growth.

**METHODS:**

We analyzed the maternal blood pressure in pregnant wild-type (*Gch1^fl/fl^*) and *Gch1^fl/fl^* Tie2cre mice treated with either FA or 5-MTHF starting before pregnancy, mid-pregnancy or late pregnancy. BH4, superoxide, and NO bioavailability were determined in mouse and human models of endothelial cell BH4 deficiency by high-performance liquid chromatography.

**RESULTS:**

In vitro studies in mouse and human endothelial cells showed that treatment with 5-MTHF, but not FA, elevated BH4 levels, reduced superoxide production, and increased NO synthase activity. In primary endothelial cells isolated from women with hypertensive pregnancies, exposure to 5-MTHF, but not FA, restored the reduction in BH4 levels and NO synthase activity. In vivo studies in mice revealed that oral treatment with 5-MTHF, but not FA, prevented and treated hypertension in pregnancy when administered either before or during pregnancy, respectively, and normalized placental and fetal growth restriction if administered from mid-gestation onward.

**CONCLUSIONS:**

Collectively, these studies identify a critical role for 5-MTHF in endothelial cell function in pregnancy, related to endothelial cell BH4 availability and NO synthase activity. Thus, 5-MTHF represents a novel therapeutic agent that may potentially improve endothelial function in hypertensive disorders of pregnancy by targeting endothelial cell BH4.

**NOVELTY AND RELEVANCE**

**What Is New?**

- We demonstrate that treatment with 5-methyl-(6S)-tetrahydrofolate (5-MTHF), but not folic acid, elevates tetrahydrobiopterin (BH4) levels, reduces superoxide production, and enhances NO synthase activity in mouse and human models of endothelial cell of BH4 deficiency.
- Treatment with 5-MTHF, but not folic acid, restores the reduction in BH4 levels and NO synthase activity in primary endothelial cells isolated from women with hypertensive pregnancies.
- Oral treatment with 5-MTHF, but not folic acid, is able to both prevent and treat progressive pregnancy-induced hypertension in mice due to endothelial cell BH4 deficiency while normalizing placental and fetal growth.

**What Is Relevant?**

- Hypertensive disorders of pregnancy are a major global health problem that affects both mothers and offspring and have long-term impacts on future cardiovascular risk in adulthood.
- This study identifies 5-MTHF as a novel potential therapeutic agent that can improve endothelial function in hypertensive disorders of pregnancy by targeting endothelial cell BH4.

**Clinical/Pathophysiological Implications?**

- The B vitamin 5-MTHF, a reduced form of folate, holds vast therapeutic potential in preventing and treating hypertensive disorders of pregnancy. Using multiple complementary in vitro and in vivo translational models, we demonstrate that 5-MTHF elevates BH4 levels, enhances endothelial NO synthase activity, and mitigates adverse effects related to endothelial cell BH4 deficiency during pregnancy. In contrast to folic acid, 5-MTHF successfully overcomes the effects of DHFR (dihydrofolate reductase) inhibition, preventing hypertension when administered before or during gestation and normalizes placental and fetal growth. The findings of this study highlight 5-MTHF as an innovative therapeutic agent for improving endothelial function in gestational hypertension and preeclampsia.

Hypertensive disorders of pregnancy, including gestational hypertension and preeclampsia, are common, affecting >80 000 women per year in the United Kingdom and millions globally.^1,2^ These disorders are associated with significant maternal and fetal morbidity and mortality. Hypertensive disorders of pregnancy are the most common cause of fetal growth restriction and premature delivery. Women with hypertensive disorders during pregnancy have a 4-fold increased risk of developing hypertension and a 2-fold increased risk of developing a stroke later in life.^3,4^ Therefore, interventions that could modify the underlying pathophysiology may have large impacts on both maternal and child health.

Maternal folate intake during pregnancy from food or food supplements is essential for fetal and placental development. Folic acid (FA) supplementation is recommended to reduce the risk of neural tube defects. A recent meta-analysis has shown that using supplements containing up to 1 mg/d FA (especially from multivitamin supplements) was associated with a lower risk of hypertensive disorders of pregnancy, including preeclampsia.^5^ However, a FACT (Folic Acid Clinical Trial) of 4 mg FA daily from week 16 until the end of pregnancy in women with risk factors for preeclampsia showed no risk reduction of preeclampsia.^5^ The active metabolite of FA is the reduced folate, 5-methyl-(6S)-tetrahydrofolate (5-MTHF), which requires sequential reduction of FA by DHFR (dihydrofolate reductase).^6^ Analysis of plasma folate levels in pregnant women receiving high-dose FA supplementation in FACT revealed a large increase in unmetabolized FA, suggesting that reduction to 5-MTHF may be limited and also explaining the lack of effect of a high dose of FA. Moreover, DHFR is also the enzyme required for the reduction of dihydrobiopterin (BH2) to tetrahydrobiopterin (BH4), a necessary cofactor for the generation of NO by eNOS (endothelial NO synthase). An excess of FA might, therefore, reduce the ability of DHFR to regenerate BH4 from BH2.

BH4 is a critical regulator of NOS (NO synthase) function and NOS-derived NO and reactive oxygen species (ROS) signaling in vascular physiology.^7,8^ Biosynthesis of BH4 is catalyzed by GTP cyclohydrolase 1, a rate-limiting enzyme for de novo BH4 biosynthesis, which is encoded by *Gch1*. We have previously shown that *Gch1* expression in endothelial cells is a key determinant of BH4 bioavailability, eNOS regulation, and thus of NO generation.^9,10^ When vascular BH4 bioavailability is limited, eNOS is unable to generate NO from l-arginine and becomes uncoupled, resulting in the generation of superoxide anion and other ROS, rather than NO.^11–14^ BH4 may have other redox-mediated effects beyond its eNOS cofactor functions, including roles in the regulation of other NOS enzymes^15^ as a cofactor for alkylglycerol monooxygenase,^16^ and NOS-independent mechanisms.^17^

eNOS is a key regulator of vascular function, with particular importance in the cardiovascular adaptation to pregnancy, through its roles in the regulation of blood flow and blood pressure (BP).^18,19^ In hypertensive pregnancy, vascular NO bioavailability is reduced and markers of oxidative stress are increased.^20,21^ We have recently demonstrated that levels of BH4 and eNOS activity are markedly reduced in primary endothelial cells and placental extracellular vesicles obtained from women with hypertensive pregnancy, compared with normotensive pregnancies.^19^ Furthermore, a mouse model of endothelial cell BH4 deficiency (the *Gch1^fl/fl^* Tie2cre mouse), generated by endothelial cell–specific deletion of *Gch1*, which encodes the BH4 biosynthetic enzyme, GTP cyclohydrolase 1, demonstrates progressive pregnancy-induced hypertension, vascular dysfunction, and intrauterine growth restriction. These findings indicate that maternal endothelial cell BH4 plays a critical role in a normal pregnancy.^18,19,22^ Oral BH4 plus 5-MTHF (but not BH4 alone) was sufficient to restore endothelial function and prevent both fetal growth restriction and hypertension in pregnant *Gch1^fl/fl^* Tie2cre mice,^18,19^ suggesting an important interaction between BH4 and folate metabolism. Furthermore, our previous studies in human vessels showed that 5-MTHF has beneficial effects on endothelial function and vascular superoxide production, by preventing peroxynitrite-mediated BH4 oxidation and improving eNOS coupling.^23^ Accordingly, we investigated whether FA and 5-MTHF have different effects on endothelial cell BH4 metabolism in pregnancy and the possible consequences for endothelial NO generation, maternal BP, and fetal growth.

## METHODS

### Data Availability

The authors declare that all supporting data are available within the article and Supplemental Material.

### Generation of Endothelial Cell-Targeted *Gch1* Knockout Mice

Endothelial cell specific BH4-deficient mice and their littermate controls were generated by crossing *Gch1^fl/fl^* females with *Gch1^fl/fl^* Tie2cre male mice as described previously.^7^ Mice were housed in individually ventilated cages (between 4 and 6 mice per cage of mixed genotypes) under specific pathogen-free conditions with a 12-hour light/dark cycle and controlled temperature (20–22 °C) and fed standard chow containing 4 mg FA per kg diet (Teklad Global 16% Protein Diet; Harlan Laboratories) and water ad libitum. Adult female *Gch1^fl/fl^* Tie2cre mice and their *Gch1^fl/fl^* littermates (thereafter referred to as wild type) on a pure (>10 generations) C57BL6/J background were bred in-house and used for all experiments at 10 to 16 weeks. The generation and phenotyping of the knockout model were conducted in accordance with the Animal (Scientific Procedures) Act 1986, with procedures reviewed by the Clinical Medicine Animal Care and Ethical Review Body, and conducted under project license (PPL) P0C27F69A. Mice were genotyped by polymerase chain reactions using DNA prepared from ear biopsies. For *Gch1^fl/fl^* genotyping, polymerase chain reaction was performed using the following primers: *Gch1^fl/fl^*-Fw 5′-GTC CTT GGT CTC AGT AAA CTT GCC AGG-3′, *Gch1^fl/fl^*-Rv 5′-GCC CAG CCA AGG ATA GAT GCA G-3′. The *Gch1* floxed allele showed a 1030 bp. For Tie2cre genotyping, polymerase chain reaction was performed using the following primers: Tie2cre Fw 5′-GCA TAA CCA GTG AAA CAG CAT TGC TG-3′, Tie2cre Rv 5′-GGA CAT GTT CAG GGA TCG CCA GGC G-3′. The Tie2cre allele amplified as a 280-bp fragment.

### Timed Mating and Treatment During Pregnancy in Mice

Pregnancy was achieved by mating either Virgin female *Gch1^fl/fl^* Tie2cre or *Gch1^fl/fl^* (wild-type) females (aged between 10 and 16 weeks old) with a *Gch1^fl/fl^* male. To evaluate the gestation day, vaginal plugs were checked for daily (morning) the presence of a plug was taken as day 0.5 of gestation (E0.5). Unless otherwise stated, all tissues were harvested and collected for experiments at either preconception (before timed mating) or E18.5 day of gestation (late gestation, 1 day before normal term delivery).

### Intervention With 5-MTHF Versus FA During Pregnancy

**Figure 1.**
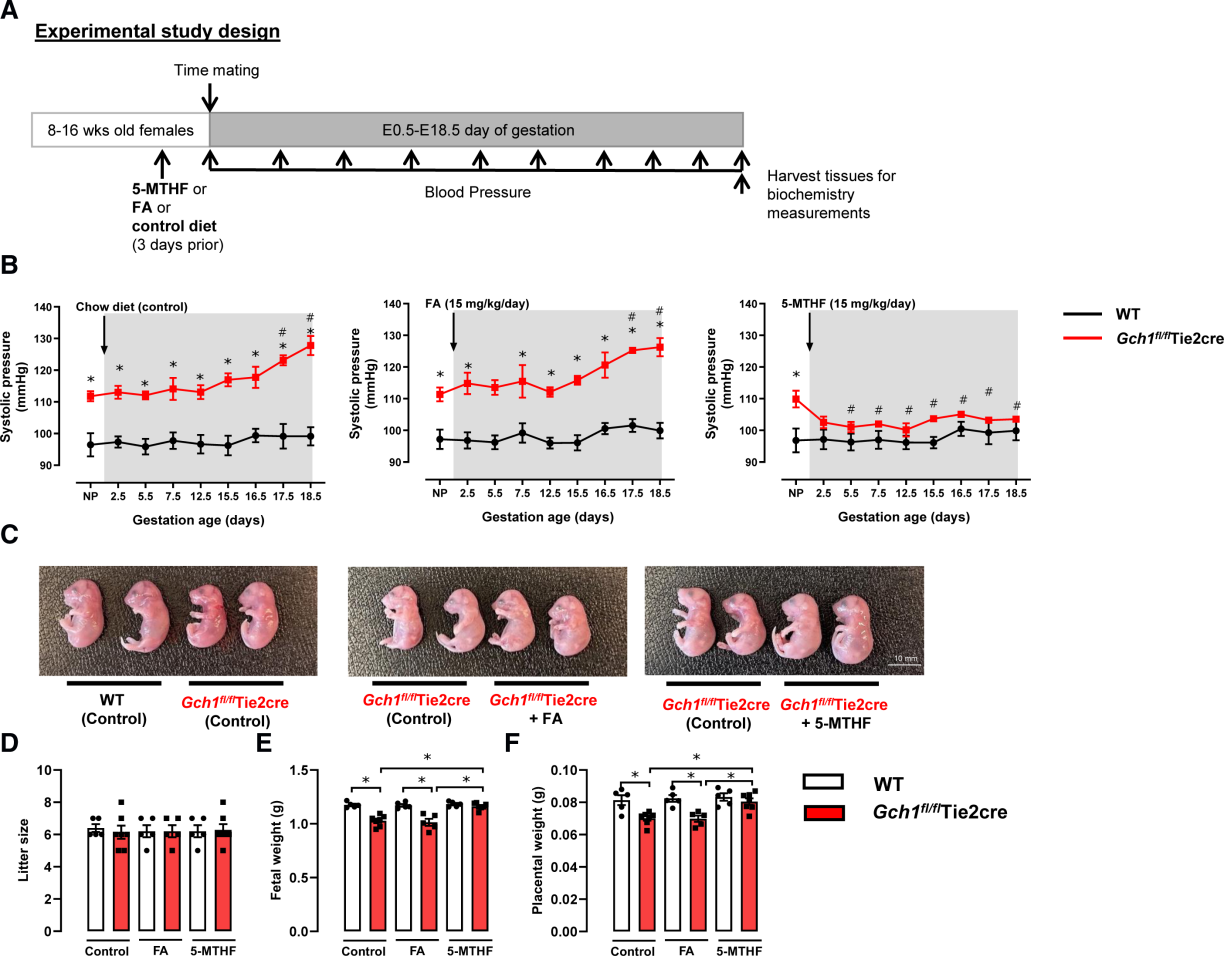
**Gestational blood pressure and fetal growth in endothelial cell tetrahydrobiopterin-deficient mice treated with either 5-methyl-(6S)-tetrahydrofolate or folic acid.** *Gch1^fl/fl^* Tie2cre and wild-type mice were treated with either oral folic acid (15 mg/kg mouse body weight/day, supplemented in chow), oral 5-methyl-(6S)-tetrahydrofolate (15 mg/kg mouse body weight/day), or control diet for 3 days before timed matings, and throughout the subsequent pregnancies. Blood pressure was determined before and throughout pregnancy by noninvasive tail-cuff plethysmography. **A**, Experimental design used in this study. **B**, Oral 5-methyl-(6S)-tetrahydrofolate treatment, but not folic acid, was sufficient to prevent progressive pregnancy-induced hypertension in *Gch1^fl/fl^* Tie2cre mice (*P<0.05, comparing genotype; #*P*<0.05, comparing to basal BP in *Gch1^fl/fl^* Tie2cre mice; n=5–7 animals per group). **C**, Representative images of fetuses were collected at E18.5 day of gestation from wild-type and *Gch1^fl/fl^* Tie2cre mice treated with either oral folic acid, **5**- methyl-(6S)-tetrahydrofolate, or control diet. **D**, The number of fetuses per litter (litter size) between wild-type and *Gch1^fl/fl^* Tie2cre mice. **E** and **F**, Fetal weight and placental weight were collected at E18.5 day of gestation (*P<0.05; n=30–50 pups from 5 to 7 litters per group). Data are shown as mean±SEM.

FA was purchased from Sigma, and the calcium salt of 5-MTHF was provided by Merck & Cie KmG. Plugged wild-type and *Gch1^fl/fl^* Tie2cre females were randomly assigned to receive normal chow (control), FA-supplemented chow (15 mg/kg mouse body weight per day), or 5-MTHF supplemented chow (15 mg/kg per mouse body weight per day), beginning 3 days before timed mating (before pregnancy), at mid (day E10.5 of gestation), or at late pregnancy (day E16.5 of gestation; see experimental study design in Figure 1A). Doses of 5-MTHF and FA were selected based on published data.^24–28^

### BP Measurement by Tail-Cuff Plethysmography

Systolic BP in conscious wild-type and *Gch1^fl/fl^* Tie2cre mice was determined using the Visitech computerized tail-cuff plethysmography system (Visitech) following 5 days of training and 3 days of consecutive baseline measurements. Experiments were performed between the hours of 8 and 12 am. The animal tails were passed through a cylindrical latex tail-cuff and taped down to reduce movement. Twenty readings were taken per mouse of which the first 5 readings were discarded. The remaining 15 readings were used to calculate the mean systolic BP in each mouse. Systolic BP of plugged *Gch1^fl/fl^* Tie2cre and wild-type mice was determined throughout gestation (embryonic days E0, E2.5, E5.5, E7.5, E10.5, E12.5, E15.5, E16.5, E17.5, and E18.5).

### Cell Culture Experiments

Human umbilical vein endothelial cells (HUVECs; Lonza) and telomerase-immortalized human aortic endothelial cells (TeloHAEC) were cultured in standard endothelial basal medium 2 (Lonza) with a bullet kit as recommended (Cat CC-3162; Lonza). All primary human endothelial cell studies were performed with cells from passage 3 to 6. Murine endothelial cells (skin-derived endothelial cells.1 [sEND]) and immortalized human vascular endothelial cells (EA.Hy926 [the human umbilical vein cell line]) were grown in standard DMEM (Invitrogen) supplemented with 10% fetal bovine serum (Sigma), glutamine (2 mmol/L; Sigma), penicillin (100 U/mL; Sigma), and streptomycin (0.1 mg/mL; Sigma). All cell cultures were maintained in humidified 5% CO_2_ at 37 °C. Cells were harvested and stored at −80 °C until used for protein and biochemistry measurements.

### BH4 and Biopterin Measurements

Intracellular concentrations of BH4, BH2, and biopterin were determined in mouse and human endothelial cells by high-performance liquid chromatography (HPLC) followed by electrochemical and fluorescence detection, respectively, following an established protocol.^9,29^ Briefly, endothelial cells were freeze-thawed in ice-cold resuspension buffer (50 mmol/L phosphate-buffered saline, 1 mmol/L dithioerythritol, 1 mmol/L EDTA, pH 7.4). After centrifugation at 13 200 rpm for 10 minutes at 4 °C, the supernatant was removed and ice-cold acid precipitation reagent (1 mmol/L phosphoric acid, 2 mol/L trichloroacetic acid, 1 mmol/L dithioerythritol) was added. Samples were vigorously mixed and then centrifuged for 5 minutes at 13 000 rpm and 4 °C. The supernatants were transferred and injected onto an isocratic HPLC system and quantified using sequential electrochemical (Coulochem III; ESA, Inc) and fluorescence (Jasco) detection. HPLC separation was performed using a 250-mm ACE C-18 column (Hichrom) and a mobile phase comprised of sodium acetate (50 mmol/L), citric acid (5 mmol/L), EDTA (48 μmol/L), and dithioerythritol (160 μmol/L; pH 5.2; all ultrapure electrochemical HPLC grade) at a flow rate of 1.3 mL/min. Background currents of +500 and −50 μA were used for the detection of BH4 on electrochemical cells E1 and E2, respectively. BH2 and B were measured using a Jasco FP2020 fluorescence detector set at 510 nm excitation and 595 nm emission. Quantification of BH4, BH2, and B was done by comparison with authentic external standards and normalized to sample protein content.

### NOS Activity Assay

eNOS activity was determined by ^14^C l-arginine to ^14^C l-citrulline conversion, followed by HPLC, in the presence and absence of an l-Arginine analog that acts as a competitive inhibitor of all 3 isoforms of NOS (N^G^-monomethyl-l-arginine; Sigma-Aldrich, United Kingdom), as described previously. Briefly, endothelial cells were seeded into 6-well culture plates (5×10^4^ cells per well) containing EGM-2 Endothelial Cell Growth Medium-2 (EBM2) medium with the bullet kit (CC-3162; Lonza; for HUVECs) or DMEM with 10% fetal bovine serum (Sigma), glutamine (2 mmol/L; Sigma), penicillin (100 U/mL; Sigma), and streptomycin (0.1 mg/mL; Sigma; for mouse endothelial cells). Cultures were maintained at 37 °C in a humidified 5% CO_2_ air atmosphere for 24 hours before measuring eNOS activity. For measuring eNOS activity, the culture media were removed before rinsing with Krebs-Hepes buffer (KHB; consisting of [in mmol/L] NaCl 99, KCl 4.7, MgSO_4_ 1.2, KH_2_PO_4_ 1.0, CaCl_2_ 1.9, NaHCO_3_ 25, glucose 11.1, and Na-Hepes 20, pH 7.40). KHB (100 μL) was then added to the cultures, or cells were preincubated with 100 μL of KHB containing 1 mmol/L of the competitive NOS inhibitor, NG-monomethyl-l-arginine, or 1 to 10 000 μM of the arginase inhibitor, Nω-Hydroxy-nor-L-arginine, Diacetate Salt (nor-NOHA; Calbiochem, United Kingdom), for 30 minutes at 37 °C. Subsequently, the KHB was replaced and 100 μL of KHB containing 1 μM calcium ionophore A23187 (Sigma-Aldrich, United Kingdom), or 1 mmol/L NG-monomethyl-l-arginine was added. Ubiquitously labeled ^14^C l-arginine (3 μL of 1.85 MBq/mL; Amersham Biosciences UK Ltd, Chalfont St. Giles, United Kingdom) was added to each well, and the cultures were incubated at 37 °C for 4 hours. The supernatants were transferred to Eppendorf tubes, and the cells were lysed by the addition of 200 μL water and freeze-thawing. The resulting lysate was added to the Eppendorf tubes containing the supernatant. Samples were deproteinated by the addition of 300 μL 10% trichloroacetic acid to the cell lysates followed by centrifugation. Endothelial cell samples were run on a Supelcosil LC-SCX 5 mm cation-exchange HPLC column (Supelco), using a Jasco HPLC system, consisting of 2 PU2080Plus pumps, a PU-2080-32 mixer, a DG-980-50 degasser, an AS2057Plus automated autosampler, a β-RAM model 3 liquid scintillation detector (Lab Logic), and Azur software (Jasco, United Kingdom). The elution gradient was generated from a mixture of 200 mmol/L citric acid pH 3.0 and MilliQ-water. To eliminate background signals, ^14^C l-arginine alone (without cells or lysate) was similarly incubated. ^14^C l-citrulline alone was also similarly incubated to determine elution times. The ^14^C l-citrulline elution peaks were integrated and expressed as a percentage of total ^14^C counts.

### Quantification of Superoxide Production

Superoxide and other ROS production were quantified by measuring the production of 2-hydroxyethidium and ethidium, respectively, produced from dihydroethidium. The quantification of 2-hydroxyethidium and ethidium was performed using methods described previously.^7^ Briefly, the endothelial cells were washed 3× with phosphate-buffered saline and then incubated for 1 hour in darkness in KHB in the presence or absence of polyethylene glycol-superoxide dismutase (100 unit/mL). After 1 hour, dihydroethidium (25 μM) was added, and the cells were then incubated for an additional 20 minutes at 37 °C. The cells were then harvested by scraping, centrifuged, and lysed in ice-cold methanol. Hydrochloric acid (100 mmol/L) was added (1:1 v/v) before loading into the autosampler for analysis. Samples were stored in darkened tubes and protected from light at all times. Separation of dihydroethidium, 2-hydroxyethidium, and ethidium was performed using a gradient HPLC system (Jasco, United Kingdom) with an ODS3 reverse-phase column (250 mm, 4.5 mm; Hichrom) and quantified using a fluorescence detector set at 510 nm (excitation) and 595 nm (emission). A linear gradient was applied from Mobile Phase A (0.1% trifluoroacetic acid) to Mobile Phase B (0.085% trifluoroacetic acid in acetonitrile) over 23 minutes (30%–50% acetonitrile).

### Clinical Studies

Mothers being cared for by Oxford University Hospitals NHS Foundation Trust between 2013 and 2015 were identified by their clinical care team and invited to take part in the Oxford Cardiovascular Tissue Bioresource program, coordinated by the Oxford Cardiovascular Clinical Research Facility and NHS Blood and Transplant, as previously described.^30–32^ Mothers and infants were recruited from normotensive pregnancies and pregnancy-induced hypertension, defined according to International Society for the Study of Hypertension in Pregnancy (ISSHP) guidelines.^33^ The exclusion criteria were the age of the mothers below 16 years and chronic cardiovascular conditions in the mother before pregnancy, including preexisting hypertension.^30^ These exclusion criteria were decided on the eligibility criteria for the clinical study to reduce variability and heterogeneity of the study participants and to minimize confounding effects. Umbilical cords were collected immediately after delivery by a dedicated research team. All cords were processed within 12 hours of delivery. HUVECs were isolated and stored in liquid nitrogen according to standard operating procedures. For the purpose of the current study, HUVECs were identified from normotensive pregnancies and pregnancy-induced hypertension, matched for maternal age and gestation. All participants provided written consent for the collection and subsequent experimental use of samples in accordance with approvals from appropriate National Research Ethics Committees (09/H0606/68, 07/H0606/148, 15/SC/0027, and 11/SC/0230).

### Folate Metabolites

Mouse endothelial cells were treated with either 5-MTHF or FA (both at 10 µmol/L), in the presence or absence of methotrexate (MTX; 1 µmol/L for 16 hours at 37 °C) to test the requirement for DHFR activity. The concentration of 5-MTHF and FA was selected according to published data.^23,34,35^ Folate metabolites were measured in cell lysates at the Department of Clinical Chemistry, Saarland University Hospital on ultra-high performance liquid chromatography (UPLC)-tandem mass spectrometry (MS/MS) using a modified sample preparation protocol. Briefly, cell pellets containing 1 million cells were used to measure folate forms. The extraction buffer (100 mmol/L ammonium acetate, 1% ascorbic acid, and 0.2% Triton X-100; pH=5.5) was added to each of the cell pellets and the mix was sonicated for 1 minute. The internal standard mix (50 µL containing 5 isotope-labeled folate forms) was added to the cell pellets, and the samples were then heated for 3 minutes at 100 °C to destroy the cell debris and proteins and then placed on ice for 5 minutes. The recombinant human gamma-glutamylhydrolase His protein (NOVUSBIO, United Kingdom; NBP1-78898 0.5 mg/1 mL) was added to the cell homogenates and samples were mixed and incubated for 1 hour at 37 °C in a water bath to enable the recombinant human gamma-glutamylhydrolase His enzyme to release folates from their polyglutamate complexes. After thorough mixing, samples were heated again at 100 °C for 3 minutes to destroy the enzyme. After centrifugation for 10 minutes at maximal *g*, the extract that contains free folates and isotope-labeled folate forms was used for the folate forms assay. Sample preparation for the folate assay was performed as reported before for serum samples.^36^ Separation of the folate forms was performed using an Acquity UPLC HSS T3 (High Strength Silica T3 Columns) (50×2.1 mm; 1.8 µm particle size) with an Acquity BEH C_18_ VanGuard precolumn (5×2.1 mm [i.d.]; 1.7 µm particle size) and a 0.2 µm in-line filter (Waters Corporation). The folate concentrations in the cell extract were expressed as nmol/million cells.

### Statistical Analysis

Data are presented as mean±SEM. Normality was tested using the D’Agostino and Pearson omnibus normality test. Groups were compared using the Mann-Whitney *U* test for nonparametric data or an unpaired Student *t* test for parametric data. When comparing multiple groups, data were analyzed by ANOVA with the Newman-Keuls posttest for parametric data or the Kruskal-Wallis test with the Dunn posttest for nonparametric data. When more than 2 independent variables were present, a 2-way ANOVA with Tukey multiple comparison test was used. When within-subject repeated measurements were present, a repeated measures ANOVA was used. A value of *P*<0.05 was considered statistically significant, and *P* values between 0.05 and 0.10 were considered indicative of a trend.

## RESULTS

### Oral 5-MTHF Treatment, but Not FA, Prevents Pregnancy-Induced Hypertension and Intrauterine Growth Restriction in Endothelial Cell BH4-Deficient Mice

We previously reported that maternal endothelial cell BH4 deficiency in *Gch1^fl/fl^* Tie2cre mice leads to hypertension and reduced fetal growth.^18,19^ We also demonstrated that oral supplementation of pregnant *Gch1^fl/fl^* Tie2cre mice with BH4 and 5-MTHF, but not BH4 alone, was sufficient to restore endothelial function and prevent both fetal growth restriction and hypertension.^18,19^ However, it is unclear whether 5-MTHF or FA alone can rescue these phenotypes associated with endothelial cell BH4 deficiency. To address this, we treated female *Gch1^fl/fl^* Tie2cre and wild-type littermates with either oral 5-MTHF (15 mg/kg mouse body weight per day) or a FA (15 mg/mouse body weight per day) or control diet (chow) for 3 days before mating and throughout pregnancy (Figure 1A).

As in our previous studies,^18,19^ maternal endothelial cell BH4 deficiency resulted in progressive hypertension and intrauterine growth restriction in pregnant *Gch1^fl/fl^* Tie2cre mice (Figure 1B through 1D). Treatment with oral FA had no significant effect on systolic BP or fetal development during pregnancy in either wild-type or *Gch1^fl/fl^* Tie2cre mice (Figure 1B through 1D). In contrast, oral 5-MTHF treatment significantly reduced systolic BP and prevented progressive hypertension and intrauterine growth restriction in *Gch1^fl/fl^* Tie2cre mice (Figure 1B through 1D). These findings indicate that, in contrast to the treatment with FA, intervention with 5-MTHF treatment before pregnancy prevented progressive hypertension and fetal growth restriction in mice with endothelial cell BH4 deficiency.

### Treatment With 5-MTHF Increases BH4 Levels in a Model of Endothelial Cells BH4 Deficiency

**Figure 2.**
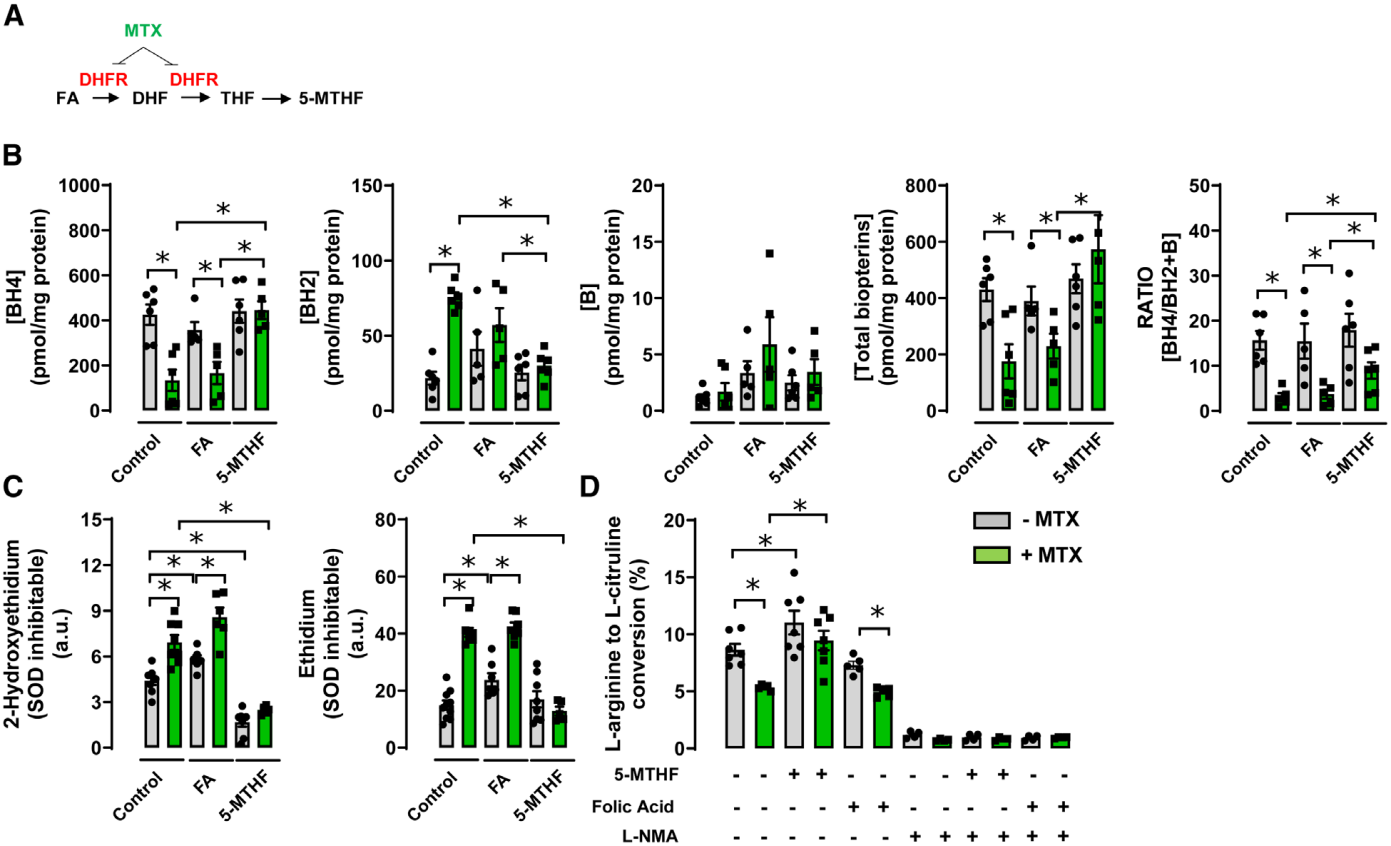
**The effect of folic acid (FA) or 5-methyl-(6S)-tetrahydrofolate (5-MTHF) on tetrahydrobiopterin levels, superoxide production, and endothelial NO synthase activity in mouse endothelial cells. A**, Schematic diagram of simplified folate metabolism. **B**, Mouse endothelial cells were treated with either FA (10 µmol/L) or 5-MTHF (10 µmol/L), in the presence or absence of methotrexate (MTX; 1 µmol/L) for 16 h at 37 °C, and intracellular biopterin levels were quantified by high-performance liquid chromatography as detailed under Methods section (*P<0.05; n=5–6 per group). **C**, Superoxide and other reactive oxygen species production were measured in mouse endothelial cells treated with either FA (10 µmol/L) or 5-MTHF (10 µmol/L) for 16 hours at 37 °C, by quantification of 2-hydroxyethidium and ethidium, respectively, using high-performance liquid chromatography. Decreased accumulation of 2-hydroxyethidium, indicative of superoxide formation, was observed in endothelial cells treated with 5-MTHF (*P<0.05; n=5–6 per group). Superoxide and reactive oxygen species productions were significantly elevated in endothelial cells treated with FA (*P<0.05; n=5–6 per group). **D**, Endothelial NO synthase activity was measured in mouse endothelial cells treated with either FA or 5-MTHF, in the presence or absence of a competitive inhibitor of NO synthase, NG-monomethyl-l-arginine (1 mmol/L) by conversion of ^14^C l-arginine in endothelial cell culture, followed by radiochemical high-performance liquid chromatography quantification of ^14^C l-citrulline production (*P<0.05; n=5–6 per group). Data are shown as mean±SEM. B indicates biopterin; BH2, dihydrobiopterin; DHF, dihydrofolate; DHFR, dihydrofolate reductase; and SOD, superoxide dismutase.

To investigate the effect of 5-MTHF versus FA on BH4 levels in endothelial cells, we treated cultured mouse endothelial cells with either 5-MTHF or FA, in the presence or absence of MTX, in order to test the requirement for DHFR activity. The effect of MTX is based on the inhibition of DHFR, which blocks the reduction of FA and, consequently, FA metabolism. BH4 levels and total biopterins were significantly decreased by exposure to MTX, whereas the levels of BH2 and biopterin were greatly increased, reflecting the requirement for DHFR in reducing BH2 to BH4 (Figure 2A and 2B). The ratio of reduced to oxidized biopterins (BH4/BH2+biopterin) was significantly decreased by MTX treatment (Figure 2B). FA supplementation had no significant effect on either BH4, BH2, biopterins, total biopterins (BH4+BH2+biopterin), or the ratio of reduced to oxidized biopterins (Figure 2A and 2B). In contrast, 5-MTHF treatment markedly elevated BH4 and total biopterins in MTX-treated endothelial cells (Figure 2A and 2B). BH2 levels were greatly reduced in MTX-treated cells compared with the control group (Figure 2B), accompanied by a significant increase in the ratio of reduced to oxidized biopterins (BH4/BH2+B; Figure 2B). These findings suggest that 5-MTHF, but not FA, is able to increase the levels of BH4 in a model of endothelial cell BH4 deficiency and can overcome the effect of MTX on inhibition of DHFR activity.

### Treatment With 5-MTHF Reduces Superoxide Productions and Increases NOS Activity in Endothelial Cell BH4 Deficiency

We next measured production of the superoxide and other ROS in mouse endothelial cells treated with either FA or 5-MTHF, by quantification of 2-hydroxyethidium and ethidium produced from dihydroethidine, using HPLC. 5-MTHF treatment significantly reduced superoxide and other ROS productions in 5-MTHF treated endothelial cells, whereas FA treatment significantly increased superoxide production (Figure 2C). eNOS activity was measured by the conversion of ^14^C l-arginine in endothelial cell culture, followed by radiochemical HPLC quantification of ^14^C l-citrulline production. As expected, eNOS activity was greatly reduced in MTX-treated endothelial cells compared with control cells (Figure 2D). 5-MTHF significantly increased eNOS activity and reversed the effect of MTX, such that eNOS activity was no longer different between the MTX-treated and control groups (Figure 2D). In contrast, FA treatment had no significant effect on eNOS activity, nor on the consequences of MTX treatment (Figure 2D). Specificity for eNOS activity was confirmed by the eNOS inhibitor NG-monomethyl-l-arginine, which abolished eNOS activity in all groups (Figure 2D). These findings indicate that treatment with 5-MTHF, but not FA, reduces superoxide production and increases eNOS activity in endothelial cells, and is able to overcome the effect of DHFR inhibition with MTX.

### FA Treatment Fails to Increase 5-MTHF Levels in Mouse Endothelial Cells

**Figure 3.**
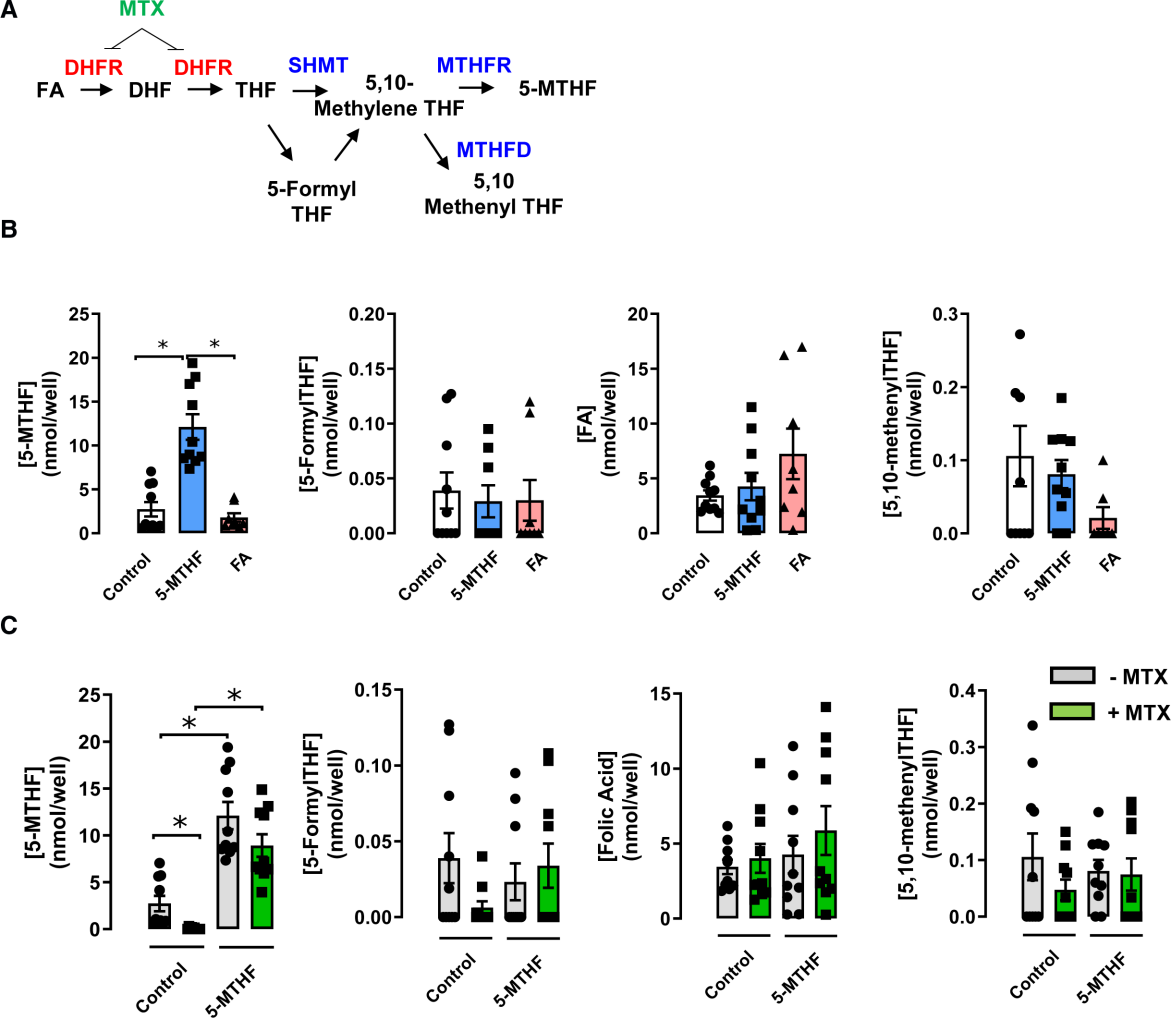
**Folate metabolites in mouse endothelial cells treated with either folic acid (FA) or 5-methyl-(6S)-tetrahydrofolate (5-MTHF). A**, Schematic diagram of simplified folate metabolism and folate species detected by liquid chromatography-tandem mass spectrometry (LC-MS/MS). **B**, The level of folate species was determined by LC-MS/MS in mouse endothelial cells treated with either folic acid (10 µmol/L) or 5-MTHF (10 µmol/L) for 16 hours at 37 °C. **C**, The level of folate species in mouse endothelial cells treated with 5-MTHF (10 µmol/L) in the presence or absence of methotrexate (1 µmol/L) for 16 hours at 37 °C (*P<0.05; n=8–9 per group). Data are shown as mean±SEM. DHF indicates dihydrofolate; DHFR, dihydrofolate reductase; and SHMT, serine hydroxymethyltransferase.

To investigate the effects of 5-MTHF and FA treatment on folate metabolites in endothelial cells, we quantified 5 folate forms (5-MTHF, FA, 5-formyl-tetrahydrofolate, and 5,10-methenyl-tetrahydrofolate) in mouse endothelial cells treated with either FA or 5-MTHF (10 µmol/L for both) by liquid chromatography (LC)-MS/MS (Figure 3A and 3B). Treatment with 5-MTHF significantly (≈2-fold) increased intracellular 5-MTHF levels in mouse endothelial cells (Figure 3B). In the FA group, there was a trend toward an increase in intracellular FA levels in FA-treated endothelial cells compared with control endothelial cells and 5-MTHF-treated endothelial cells (Figure 3B). However, intracellular 5-MTHF levels were comparable between FA-treated endothelial cells and control endothelial cells (Figure 3B), indicating that FA treatment is unable to increase 5-MTHF levels in cultured endothelial cells.

We next determined the effect of DHFR inhibition on folate species in endothelial cells exposed to MTX, treated with or without 5-MTHF (Figure 3C). We found that intracellular levels of 5-MTHF were dramatically reduced in MTX-treated endothelial cells, but this effect of MTX was completely overcome by 5-MTHF treatment (Figure 3C). Treatment with MTX had no significant effect on FA, 5-formyl-tetrahydrofolate levels, or 5,10-methenyl-tetrahydrofolate levels in endothelial cells treated with or without 5-MTHF (Figure 3C).

### Treatment With 5-MTHF Increases BH4 Levels, Reduces Reactive Oxygen Species, and Increases eNOS Activity in Primary Human Umbilical Vein Endothelial Cells

**Figure 4.**
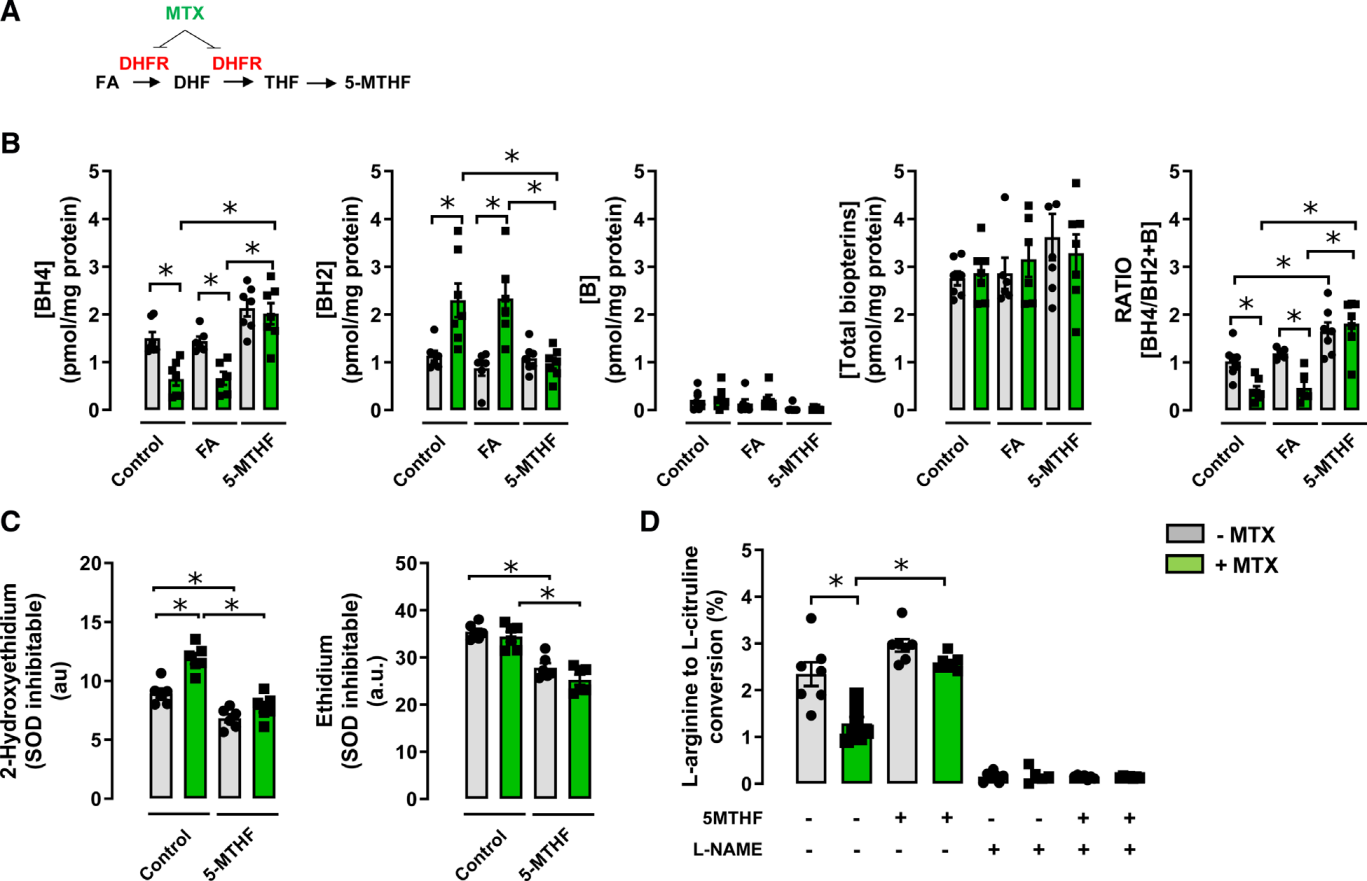
**The effect of folic acid (FA) or 5-methyl-(6S)-tetrahydrofolate (5-MTHF) on tetrahydrobiopterin, superoxide production, and endothelial NO activity in human endothelial cells. A**, Schematic diagram of simplified folate metabolism. **B**, Human umbilical vein cells from normotensive women were treated with either folic acid (10 µmol/L) or 5-MTHF (10 µmol/L), in the presence or absence of methotrexate for 16 hours at 37 °C, and intracellular biopterin levels were quantified by high-performance liquid chromatography as detailed under Experimental Procedures (*P<0.05; n=6–7 per group). **C**, Superoxide and other reactive oxygen species production in human umbilical vein cells treated with either 5-MTHF or control, in the presence or absence of methotrexate for 16 hours at 37 °C were measured by quantification of 2-hydroxyethidium and ethidium, respectively, produced from dihydroethidine, using high-performance liquid chromatography (*P<0.05; n=6 per group). **D**, Endothelial NO synthase activity in human endothelial cells treated with either 5-MTHF or control, in the presence or absence of a competitive inhibitor of NO synthase, NG-monomethyl-l-arginine (1 mmol/L) by conversion of ^14^C l-arginine in endothelial cell culture, followed by radiochemical high-performance liquid chromatography quantification of ^14^C l-citrulline production (*P<0.05; n=7 per group). Data are shown as mean±SEM. DHF indicates dihydrofolate; and DHFR, dihydrofolate reductase.

To investigate the translational relevance of these observations in mouse endothelial cells, we next tested the effect of 5-MTHF on the production of BH4, ROS, and eNOS activity in primary HUVECs, which contain low endogenous levels of BH4 that is limiting for eNOS activity.^19^ Using cultures of pooled HUVECs from healthy donor cords, we found that BH4 and total biopterin levels were significantly reduced in cells treated with MTX but significantly increased in cells treated with 5-MTHF, in both control and MTX-treated HUVECs. In contrast, FA treatment had no significant effect on BH4 or total biopterin levels in either control HUVECs or MTX-treated HUVECs (Figure 4A and 4B). Superoxide and ROS production, as determined by DHE-HPLC, were significantly increased in MTX-treated HUVECs (Figure 4C) and were decreased by 5-MTHF treatment (Figure 4C). Similar effects with 5-MTHF treatment were observed on HUVEC eNOS activity (Figure 4D).

**Figure 5.**
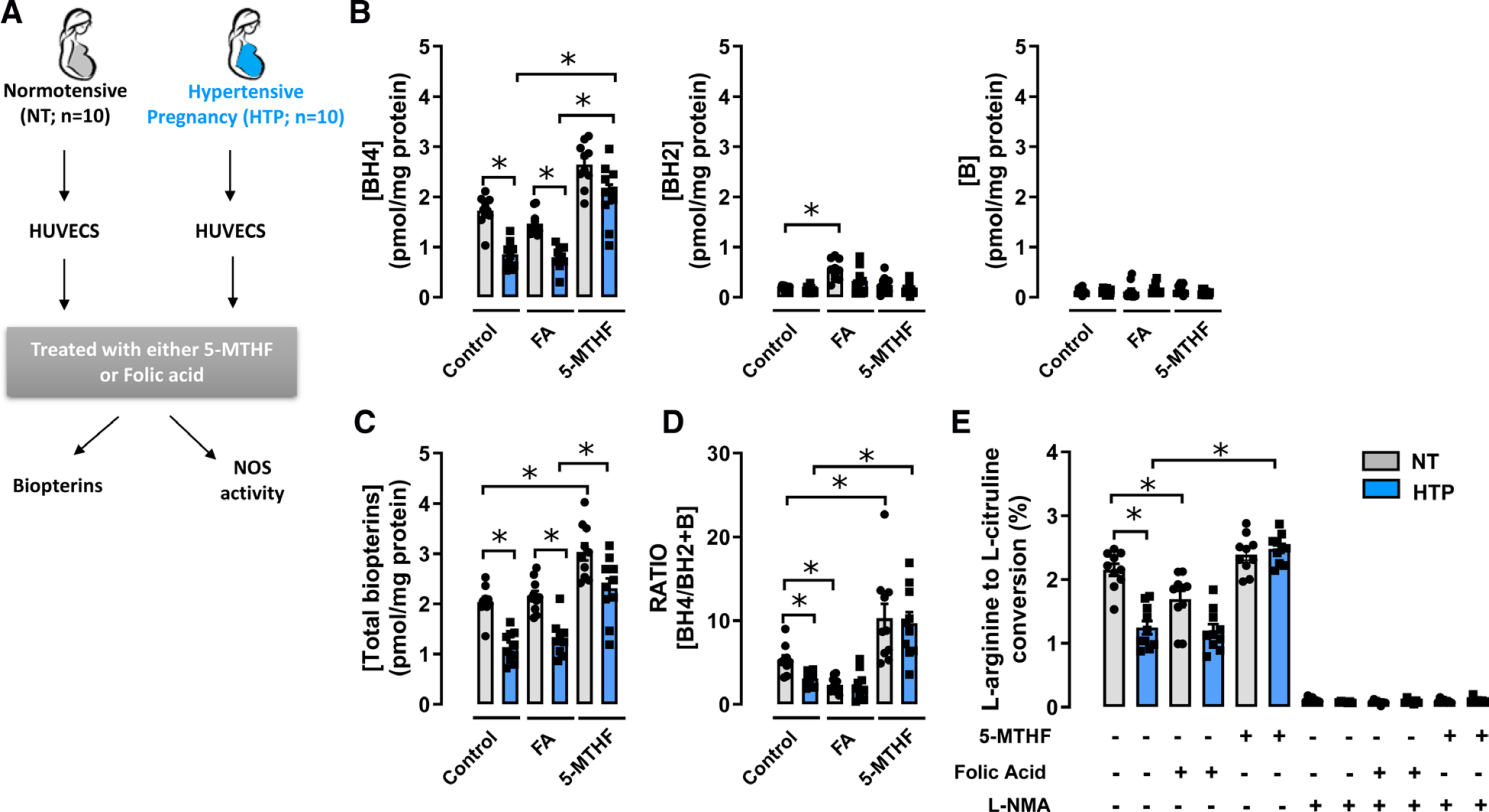
**The effect of folic acid (FA) or 5-methyl-(6S)-tetrahydrofolate (5-MTHF) treatment on tetrahydrobiopterin and endothelial NO synthase (NOS) activity in endothelial cells isolated from normotensive (NT) and hypertensive pregnancies. A**, Schematic diagram showing human umbilical vein endothelial cell (HUVEC) were isolated from umbilical cords of placentas from either NT or hypertensive pregnancies. HUVEC were treated with either FA (10 µmol/L) or 5-MTHF (10 µmol/L), in the presence or absence of methotrexate for 16 hours at 37 °C, and intracellular biopterin levels and endothelial NOS activity were quantified by high-performance liquid chromatography as detailed under Methods section (*P<0.05; n=10 per group). **B** through **D**, High-performance liquid chromatography analysis of biopterins in HUVEC from NT and hypertensive pregnancies treated with either folic acid or 5-MTHF (*P<0.05; n=10 per group). **E**, Endothelial NOS activity in human endothelial cells treated with either FA or 5-MTHF, control, in the presence, or absence of a competitive inhibitor of NOS, NG-monomethyl-l-arginine (1 mmol/L) by conversion of ^14^C l-arginine in endothelial cell culture, followed by radiochemical high-performance liquid chromatography quantification of ^14^C l-citrulline production (*P<0.05; n=10 per group). Data are shown as mean±SEM.

In line with previous results,^19^ we next investigated the effects of 5-MTHF treatment on endothelial cells cultured from individual cords, obtained from either normotensive or hypertensive pregnancies (Figure 5A). The clinical characteristics of the study participants are shown in Table S1. We found that BH4 and total biopterin levels were significantly decreased in HUVECs from hypertensive pregnancies compared with those from normotensive pregnancies, associated with a reduction in the BH4/BH2+B ratio (Figure 5B through 5D). Treatment with 5-MTHF significantly increased BH4, total biopterins, and the BH4/BH2+B ratio in endothelial cells from both normotensive and hypertensive pregnancies, such that BH4 and total biopterin levels in hypertensive pregnancy were comparable to the normotensive controls. In contrast, FA had no significant effect on either BH4 or total biopterin levels.

Furthermore, eNOS activity, measured as l-arginine to l-citrulline conversion, was decreased in HUVECs from hypertensive pregnancies compared with normotensive pregnancies (Figure 5E). Treatment with 5-MTHF significantly increased eNOS activity in HUVECSs from hypertensive pregnancies, such that eNOS activity was comparable between normotensive and hypertensive pregnancies. Treatment with FA has no significant effect on eNOS activity in HUVECs from either normotensive or hypertensive pregnancies, although there was a trend toward a decrease in NOS activity in FA-treated HUVECs.

### Treatment With 5-MTHF at Mid-Pregnancy Prevents Progressive Pregnancy-Induced Hypertension and Intrauterine Growth Restriction in Endothelial Cell-Specific BH4 Deficient Mice

**Figure 6.**
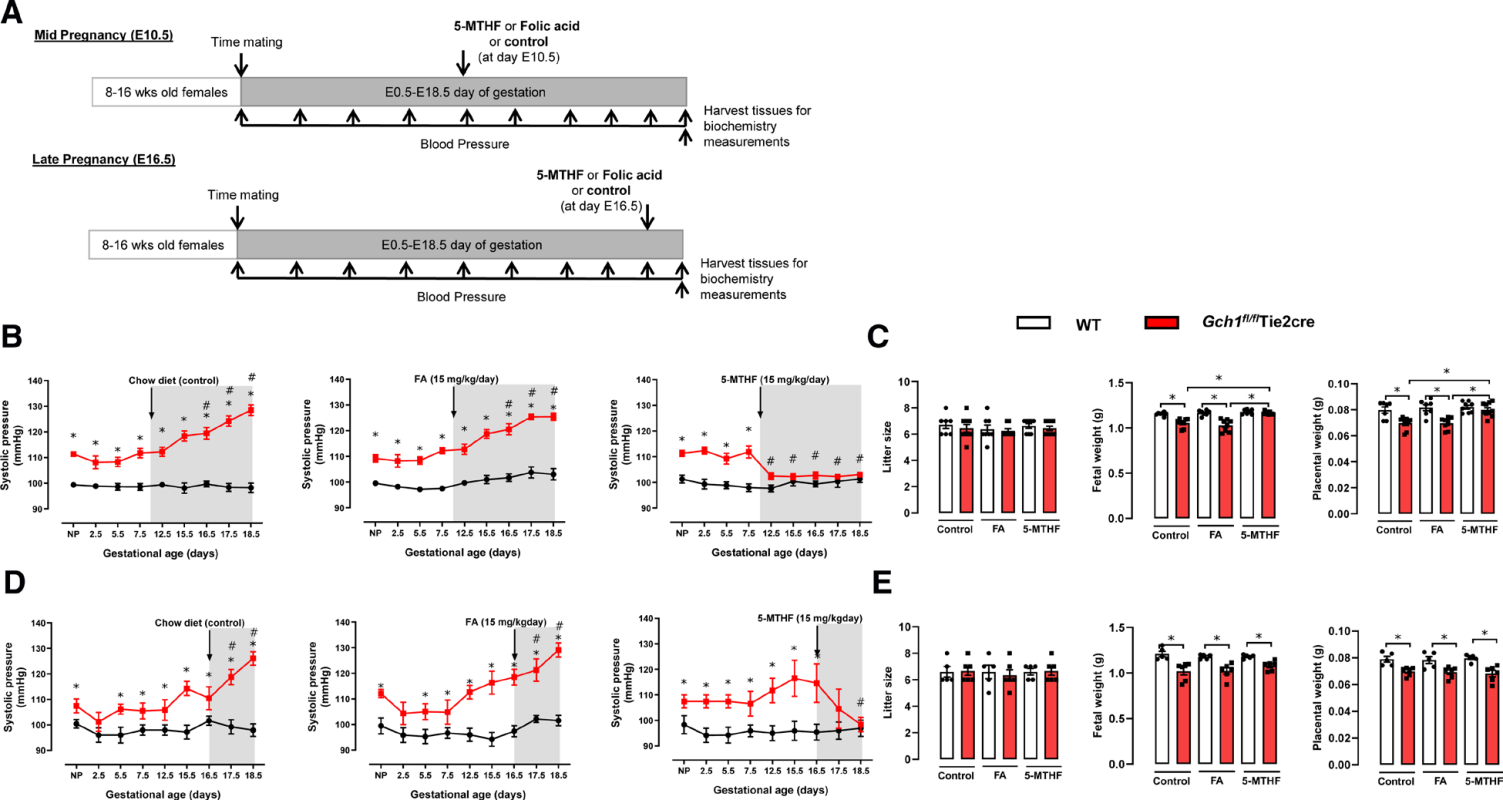
**Gestational blood pressure and intrauterine growth in endothelial cell tetrahydrobiopterin-deficient mice treated with either 5-methyl-(6S)-tetrahydrofolate (5-MTHF) or folic acid (FA) at mid- and late gestation. A**, Experimental design for the study. Pregnant *Gch1^fl/fl^* Tie2cre and wild-type (WT) mice were treated with either oral FA (15 mg/kg mouse body weight/day, supplemented in chow), oral 5-MTHF (15 mg/kg mouse body weight/day), or control diet staring at day 10.5 (mid-gestation) or day 16.5 (late gestation). Blood pressure was determined before and throughout pregnancy by noninvasive tail-cuff plethysmography. **B**, Oral 5-MTHF treatment, but not FA, at mid-gestation (E10.5) was sufficient to prevent hypertensive pregnancy in *Gch1^fl/fl^* Tie2cre mice (*P<0.05, comparing genotype; #*P*<0.05, comparing to basal blood pressure in *Gch1^fl/fl^* Tie2cre mice; n=7–9 animals per group). **C**, The number of fetuses per litter (litter size) was not different between the groups (n=7–9 animals per group). Fetal weight and placental weight were collected at E18.5 day of gestation from WT and *Gch1^fl/fl^* Tie2cre mice treated with either FA, 5-MTHF, or control diet (*P<0.05; n=40–65 pups from 7 to 9 litters per group). **D**, Oral 5-MTHF treatment, but not folic acid, at late gestation was sufficient to prevent progressive pregnancy-induced hypertension (*P<0.05, comparing genotype; #*P*<0.05, comparing to basal BP in *Gch1^fl/fl^* Tie2cre mice; n=5–6 animals per group) but not (**E**) fetal growth restriction in *Gch1^fl/fl^* Tie2cre mice (*P<0.05; n=25–35 pups from 5 to 6 litters per group). Data are shown as mean±SEM.

We next tested the effect of oral 5-MTHF or FA provided through the diet (15 mg/kg mouse weight per day) as a treatment for high BP at either mid- or late gestation in pregnant endothelial cell specific BH4-deficient mice (Figure 6A). Systolic BP in pregnant endothelial cell BH4-deficient mice was markedly elevated by day 16.5 post coitus and increased further at 18.5 days. In pregnant endothelial cell BH4-deficient mice treated with FA, systolic BP remained elevated throughout pregnancy, whereas 5-MTHF treatment normalized the elevated systolic BP when administered at either mid- or late gestation (Figure 6B and 6C). The reduction in placental and fetal weights in pregnant endothelial cell BH4-deficient mice was normalized by treatment with 5-MTHF, but not by FA, when treatment was initiated in mid-gestation (from day 10.5; Figure 6D). However, 5-MTHF treatment from late gestation (day 16.5) did not normalize placental or fetal growth, despite the reduction in BP (Figure 6E).

## DISCUSSION

In this study, we demonstrate that the reduced folate, 5-MTHF, has striking effects on endothelial cell BH4 levels and eNOS activity and is able to prevent and treat the adverse consequences of endothelial cell BH4 deficiency in pregnancy, which are not observed with FA. The key findings are as follows: (1) treatment with 5-MTHF elevates BH4 levels, reduces superoxide production, and increases eNOS activity in both mouse and human endothelial cells, and restores the reduction in BH4 levels and eNOS activity in primary endothelial cells isolated from women with hypertensive pregnancies; (2) 5-MTHF treatment is able to overcome the adverse effects of MTX-induced DHFR inhibition on endothelial cell BH4 levels and specific folate species, whereas FA does not; and (3) oral treatment with 5-MTHF, but not FA, is able to both prevent and treat when administered to mice either before or during pregnancy, respectively, and prevents placental and fetal growth restriction if administered from, at the latest, mid-gestation onward. Collectively, these studies identify a critical role for 5-MTHF in endothelial cell function in pregnancy, related to endothelial cell BH4 availability and eNOS activity.

We have previously reported that BH4 levels and eNOS activity are markedly reduced in HUVECs from women with hypertensive pregnancies,^19^ and we created a novel model of maternal endothelial cell BH4 deficiency in pregnancy using a mouse with an endothelial cell–specific deletion of *Gch1*, the gene encoding the required BH4 biosynthetic enzyme, GTP cyclohydrolase 1. Although the adverse maternal and fetal consequences of endothelial cell BH4 deficiency could not be overcome with oral BH4 supplementation, a combination of both BH4 and 5-MTHF normalized BP and both placental and fetal growth. Accordingly, we reasoned that 5-MTHF has effects on BH4 availability, potentially by preventing the oxidation of BH4, forming the inactive compounds BH2 and B, or effects via the enzyme DHFR, which functions to reduce FA and dihydrofolate to tetrahydrofolate that is further metabolized to folate cofactors, such as 5-MTHF, and also reduces BH2 to regenerate BH4. We tested the requirement for DHFR activity by exposing mouse and human endothelial cells to MTX and by comparing the effects of 5-MTHF with FA. 5-MTHF was able to increase or restore BH4 levels and eNOS activity, reduce superoxide production, and overcome the effects of DHFR inhibition, whereas FA was not. Indeed, FA tended to accentuate some consequences of DHFR inhibition, such as increasing BH2 levels and hence reducing the BH4/BH2+B ratio in primary human endothelial cells.

The importance of DHFR in maintaining endothelial cell BH4 availability was first demonstrated by Chalupsky and Cai^37^ DHFR protein was downregulated by H_2_O_2_ resulting from increased endothelial cell ROS generation. More recent studies provided proof of principle that reversal of DHFR downregulation can improve BH4-mediated effects.^38^ The independence of 5-MTHF effects on DHFR activity supports the notion that 5-MTHF acts downstream of the steps required for the reduction of FA and dihydrofolate. This may also be relevant to the role of DHFR in the reduction of BH2, generated by the oxidation of BH4, back to active BH4 that can support eNOS activity and other putative BH4 functions. Inhibition of DHFR activity by MTX is known to diminish intracellular BH4 and increased BH2 levels, resulting in enzymatic uncoupling of eNOS in endothelial cells, increased oxidative stress thus endothelial cell dysfunction.^29,39,40^ Previous studies indicate that the requirement for DHFR in maintaining adequate BH4 levels relative to BH2 is particularly marked when absolute levels of BH4 are low,^29^ providing a potential explanation for the beneficial effects of 5-MTHF in pregnancy, where endothelial cell BH4 levels are low, and specifically in a mouse model of pregnancy-associated hypertension caused by selective endothelial cell BH4 deficiency.

The beneficial effects of oral 5-MTHF treatment on maternal BP in endothelial cell BH4-deficient mice were observed shortly after administration either before and throughout pregnancy, or from mid- or late gestation onward, and occurred within 1 day of 5-MTHF initiation. These observations suggest that the drivers of high BP in pregnant endothelial cell BH4-deficient mice can be rescued rapidly, suggesting a metabolic effect on BH4 in endothelial cells. In contrast to the effect on BP, oral 5-MTHF treatment only rescued fetal growth reduction when administered either before and throughout pregnancy or from mid-gestation onward, reflecting the requirement for longer-term effects on vascular and placental function to impact fetal growth. In addition, we found that 5-MTHF treatment reduced BP in nonpregnant endothelial cell BH4-deficient mice (Figure S1) but did not further reduce systolic BP in nonpregnant wild-type mice. Taken together, these findings indicate that treatment with 5-MTHF can reduce BP in nonpregnant *Gch1^fl/fl^* Tie2cre mice as well as during gestation in *Gch1^fl/fl^* Tie2cre mice.

We have previously demonstrated that 5-MTHF has beneficial effects on endothelial cell function and vascular superoxide production in human atherosclerosis, by preventing peroxynitrite-mediated BH4 oxidation and improving eNOS coupling.^23^ Other studies have also reported that 5-MTHF had a direct beneficial effect on endothelial function, independent of plasma homocysteine levels.^41^ We reproduced the effects of 5-MTHF, but not FA, in primary endothelial cells from both pooled donors and individual pregnancies, and in several endothelial cell lines, including sEND, EA.hy926, and Telo HAEC1 (Figures S2 and S3). It is possible that 5-MTHF could act as an antioxidant that either protects BH4 from oxidation to BH2 or promotes the reduction of BH2 to regenerate BH4. However, treatment of endothelial cells with sodium ascorbate had no significant effect on BH4 levels, ROS production, or eNOS activity, nor did it alter the response to MTX exposure (Figures S4 through S6), making simple antioxidant effects less likely.

The effects of FA on endothelial cell function and cardiovascular disease are inconsistent and may be confounded by effects related to dose and treatment duration, by the reliance of related biomarkers such as plasma homocysteine to interpret FA effects, and incomplete understanding of the effects of FA treatment on specific folate species. High doses of FA can lead to a rapid saturation or inhibition of the DHFR enzyme, leading to an accumulation of unmetabolized FA. Unmetabolized FA can be detected in serum after a supplementation period of 14 weeks at 400 µg FA supplementation. The biological effects of unmetabolized FA are unknown. FA has been reported to impair the uptake of 5-MTHF in HUVECs.^42^ A randomized clinical trial of FA (4 mg/d) in more than 2400 pregnant women at risk for preeclampsia showed no clinical benefit^43^ and subsequent analysis of plasma folates in women who had received high-dose FA revealed only a modest increase in plasma 5-MTHF levels, whereas circulating levels of unmetabolized FA were increased 2-fold.^44^ Inhibition of endothelial cell 5-MTHF uptake by FA would limit 5-MTHF intracellular availability, whereas 5-MTHF supplementation would overcome any inhibitory effect of FA in either culture medium or diet that routinely contain FA.

## PERSPECTIVES

We describe new findings on the effect of 5-MTHF and FA on the prevention and treatment of pregnancy-related hypertension, a major global health problem that affects both mothers and offspring, and has long-term impacts on future cardiovascular risk in adulthood. Using a targeted mouse model of endothelial cell deficiency of BH4, a required cofactor for NOS activity, we now demonstrate that 5-MTHF, but not FA, has beneficial effects on endothelial cell BH4/eNOS function and is able to prevent and treat progressive pregnancy-induced hypertension in mice. In primary endothelial cells isolated from women with hypertensive pregnancies, treatment with 5-MTHF, but not FA, restores the reduction in BH4 levels and NOS activity. Collectively, these studies identify a critical role for 5-MTHF in endothelial cell function in pregnancy, related to endothelial cell BH4 availability and NOS activity. Thus, 5-MTHF represents a novel therapeutic agent that may potentially improve endothelial function in hypertensive disorders of pregnancy by targeting endothelial cell BH4.

## ARTICLE INFORMATION

### Author Contributions

Conceptualization: K.M. Channon, R. Boehni, R. Moser, G. Douglas, and S. Chuaiphichai. Data curation: Y. Dickinson, A.J. Lewandowski, and S. Chuaiphichai. Formal analysis: Y. Dickinson, A.J. Lewandowski, and S. Chuaiphichai. Funding acquisition: K.M. Channon and S. Chuaiphichai. Investigation: Y. Dickinson, R. Boehni, R. Obeid, J.-P. Knapp, R. Moser, A.J. Lewandowski, G. Douglas, P. Leeson, K.M. Channon, and S. Chuaiphichai. Methodology: Y. Dickinson, R. Boehni, R. Obeid, J.-P. Knapp, R. Moser, G. Douglas, and S. Chuaiphichai. Supervision: G. Douglas, P. Leeson, K.M. Channon, and S. Chuaiphichai. Writing and original draft: Y. Dickinson, K.M. Channon, and S. Chuaiphichai. Writing review and editing: Y. Dickinson, R. Boehni, R. Obeid, J.-P. Knapp, R. Moser, A.J. Lewandowski, G. Douglas, P. Leeson, K.M. Channon, and S. Chuaiphichai.

### Sources of Funding

This study was supported by British Heart Foundation (BHF) program grants (RG/17/10/32859 and RG/F/22/110085), BHF Project grant (PG/19/48/34433), BHF Chair award (CH/16/1/32013), Oxford BHF Centre of Research Excellence (RE/13/1/30181), and Merck & Cie KmG, Schaffhausen, Switzerland.

### Disclosures

None.

## Supplementary Material


