# Supplementary material for: Novel Role of 5-Methyl-(6S)-Tetrahydrofolate in Mediating Endothelial Cell Tetrahydrobiopterin in Pregnancy and Implications for Gestational Hypertension
Source: Hypertension. 2024 Jul 23;81(9):1910–23. doi: 10.1161/HYPERTENSIONAHA.124.22838 (PMC11319083; doi:10.1161/HYPERTENSIONAHA.124.22838)
Supplement: Supplementary file 1 [file hyp-81-1910-s001.pdf]

Supplemental Material

A Novel Role of 5-Methyl-(6S)-tetrahydrofolate in Mediating Endothelial Cell Tetrahydrobiopterin in Pregnancy and Implications for Gestational Hypertension

Running Title: *Dickinson et al*; *5-MTHF effect on BH4 and Hypertensive Pregnancy*

Yasmin Dickinson[1], Ruth Boehni[4], Rima Obeid[5], Jean-Pierre Knapp[4], Rudolf Moser[4], Adam J. Lewandowski[3], Gillian Douglas[1], Paul Leeson[2], Keith M. Channon[1 *], Surawee Chuaiphichai[1 *]

[1]Division of Cardiovascular Medicine, British Heart Foundation Centre of Research Excellence, Radcliffe Department of Medicine, University of Oxford, Oxford, OX3 9DU, UK

[2]Oxford Cardiovascular Clinical Research Facility, Division of Cardiovascular Medicine, Radcliffe Department of Medicine, University of Oxford, Oxford OX3 9DU, UK

[3]Nuffield Department of Population Health, University of Oxford, Oxford OX3 7LF, UK

[4]Merck & Cie KmG, Im Laternenacker 5, CH-8200 Schaffhausen, Switzerland

[5]Department of Clinical Chemistry and Laboratory Medicine, Saarland University Hospital, D-66424 Homburg/Saar, Germany.

[*]Corresponding authors:

Surawee Chuaiphichai, DPhil
Division of Cardiovascular Medicine
British Heart Foundation Centre of Research Excellence,
Radcliffe Department of Medicine,
University of Oxford,
Oxford, OX3 9DU, UK
Tel: +44(0)1865 287662
e-mail: surawee.chuaiphichai@cardiov.ox.ac.uk

Keith M.Channon, FMedSci FRCP, MD
Division of Cardiovascular Medicine
British Heart Foundation Centre of Research Excellence,
Radcliffe Department of Medicine,
University of Oxford,
Oxford, OX3 9DU, UK
Tel: +44(0)1865 572783
e-mail: keith.channon@cardiov.ox.ac.uk

Table S1. Characteristics of Cohort (HUVECs)

Parameters	Normotensive (n=10)	Hypertensive (n=10)	P-value
**Maternal**			
**Maternal age, years**	34.9±3.1	35.0±3.2	0.78
**BMI at booking, kg/m²**	22.2±3.5	28.6±5.2	0.004
**Smokers, n(%)**	0(0)	1(10)	0.31
**LFT Abnormalities, n(%)**	0(0)	4(40)	0.018
**Booking SBP, mmHg**	109.7±9.8	119.7±7.5	0.003
**Booking DBP, mmHg**	67.8±5.7	75.2±9.2	0.046
**Late gestation SBP, mmHg**	111.0±9.5	124.4±11.3	0.012
**Late gestation DBP, mmHg**	66.9±8.6	78.4±8.5	0.005
**Highest SBP, mmHg**	117.6±5.6	143.7±5.5	<0.001
**Highest DBP, mmHg**	73.0±9.3	85.8±9.9	0.002
**Fetal**			
**Gestational age, weeks**	39.3±1.5	39.3±1.2	0.90
**Males, n(%)**	4(40)	3(30)	0.36
**Birthweight, grams**	3285±513	3398±391	0.87
**SGA, n(%)**	0(0)	0(0)	>0.99
**Head circumference, mm**	172.9±17.0	176.4±8.0	0.63
**Abdominal circumference, mm**	150.2±18.0	155.3±9.7	0.88
**Femur length, mm**	32.6±4.5	32.6±1.5	0.25

Values as Mean ± Standard Deviation unless stated otherwise.
sBP systolic blood pressure; dBP diastolic blood pressure; LFT; liver function test.

Figure S1

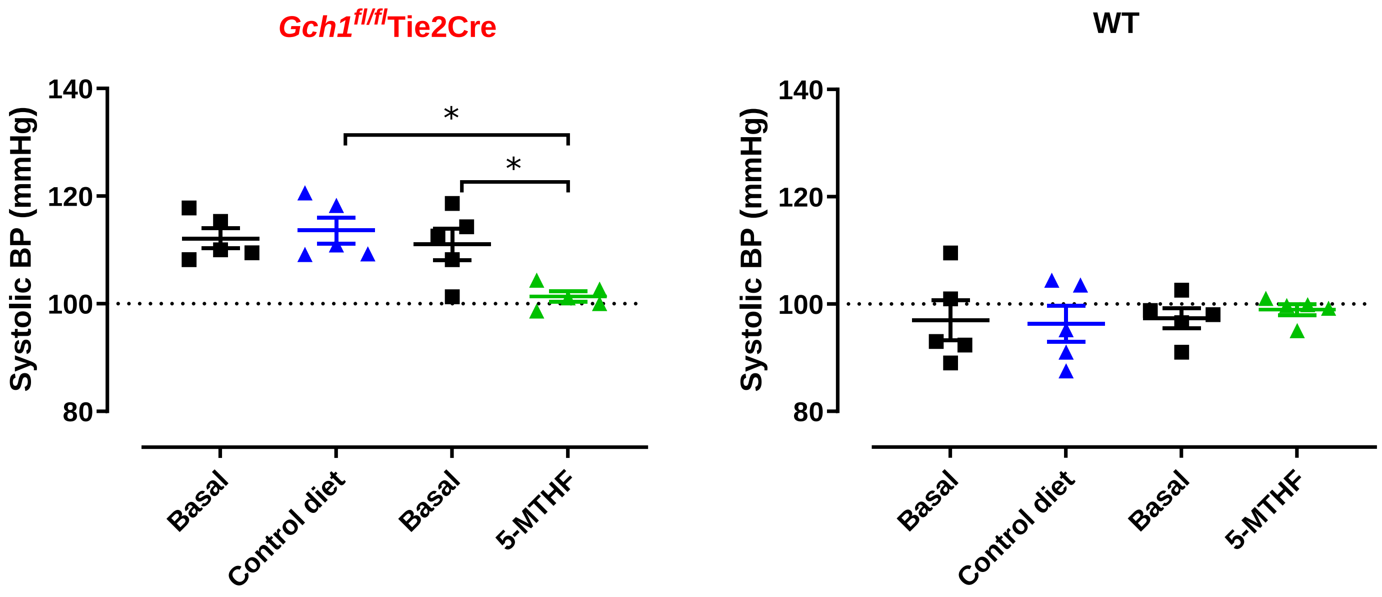

Figure S1. Blood pressure in non-pregnant endothelial cell BH4 deficient mice treated with 5-methyltetrahydrofolate (5-MTHF). Experimental design for the study. Non-pregnant *Gch1*$^{fl/fl}$Tie2cre and wild-type (WT) mice were treated with oral 5-MTHF (15 mg/kg mouse body weight/day) or control diet for 7 days. Blood pressure was determined by non-invasive tail-cuff plethysmography (*$P < 0.05$; $n = 5$ animals per group). Data are shown as means ± S.E.M.

Figure S2

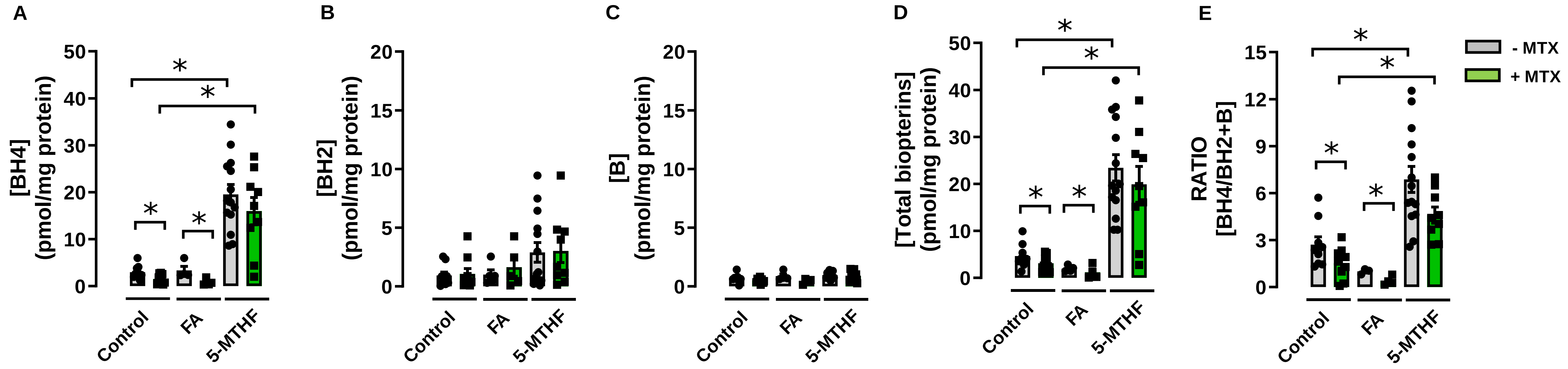

Figure S2. The effect of folic acid (FA) or 5-methyltetrahydrofolate (5-MTHF) on BH4 levels in EA.hy926 endothelial cells.
(**A-E**) EA.hy926 cells are immortalized endothelial cell line which are a hybrid cell line that results from the fusion of HUVECs and A549 lung carcinoma cells. EA.hy926 cells were treated with either FA (10 µM) or 5-MTHF (10 µM), in the presence or absence of methotrexate (MTX; 1 µM) for 16 h at 37°C, and intracellular biopterin levels were quantified by HPLC as detailed under Experimental Procedures. (*$P < 0.05$; n=5 to 9 per group).

Figure S3

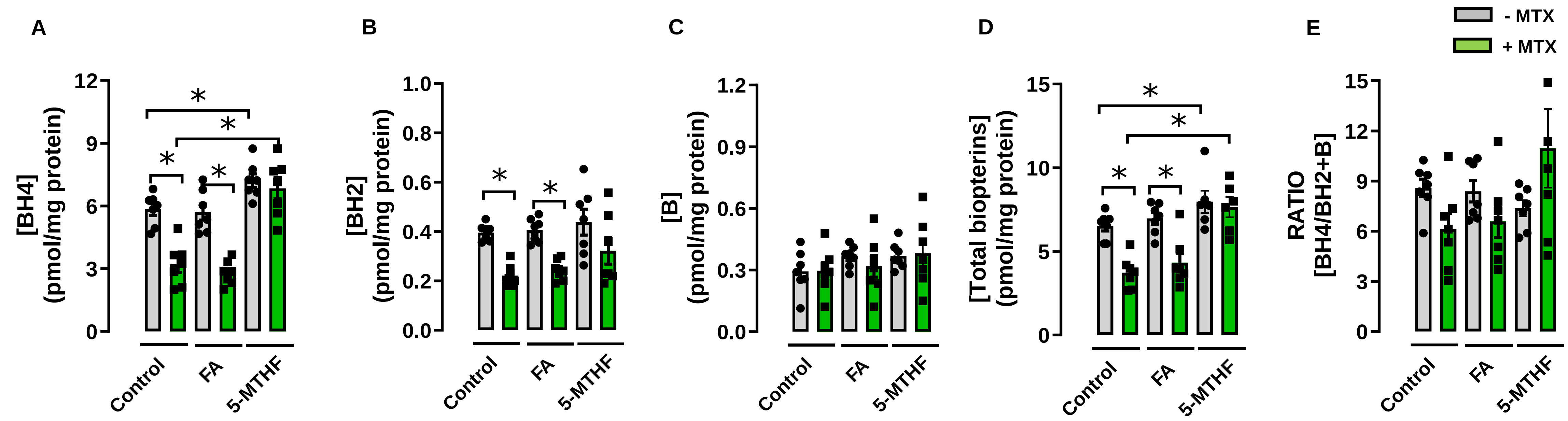

Figure S3. The effect of folic acid (FA) or 5-methyltetrahydrofolate (5-MTHF) on BH4 levels in TeloHAEC endothelial cells.
(**A-E**) TeloHAEC are immortalized human aortic endothelial cells with many TeloHAEC is a clonal cell line immortalized by stably expressing human telomerase catalytic subunit hTERT in HAEC. TeloHAEC cells were treated with either FA (10 µM) or 5-MTHF (10 µM), in the presence or absence of methotrexate (MTX; 1 µM) for 16 h at 37°C, and intracellular biopterin levels were quantified by HPLC as detailed under Experimental Procedures. (*$P < 0.05$; n=7 to 8 per group). Data are shown as means ± S.E.M.

Figure S4

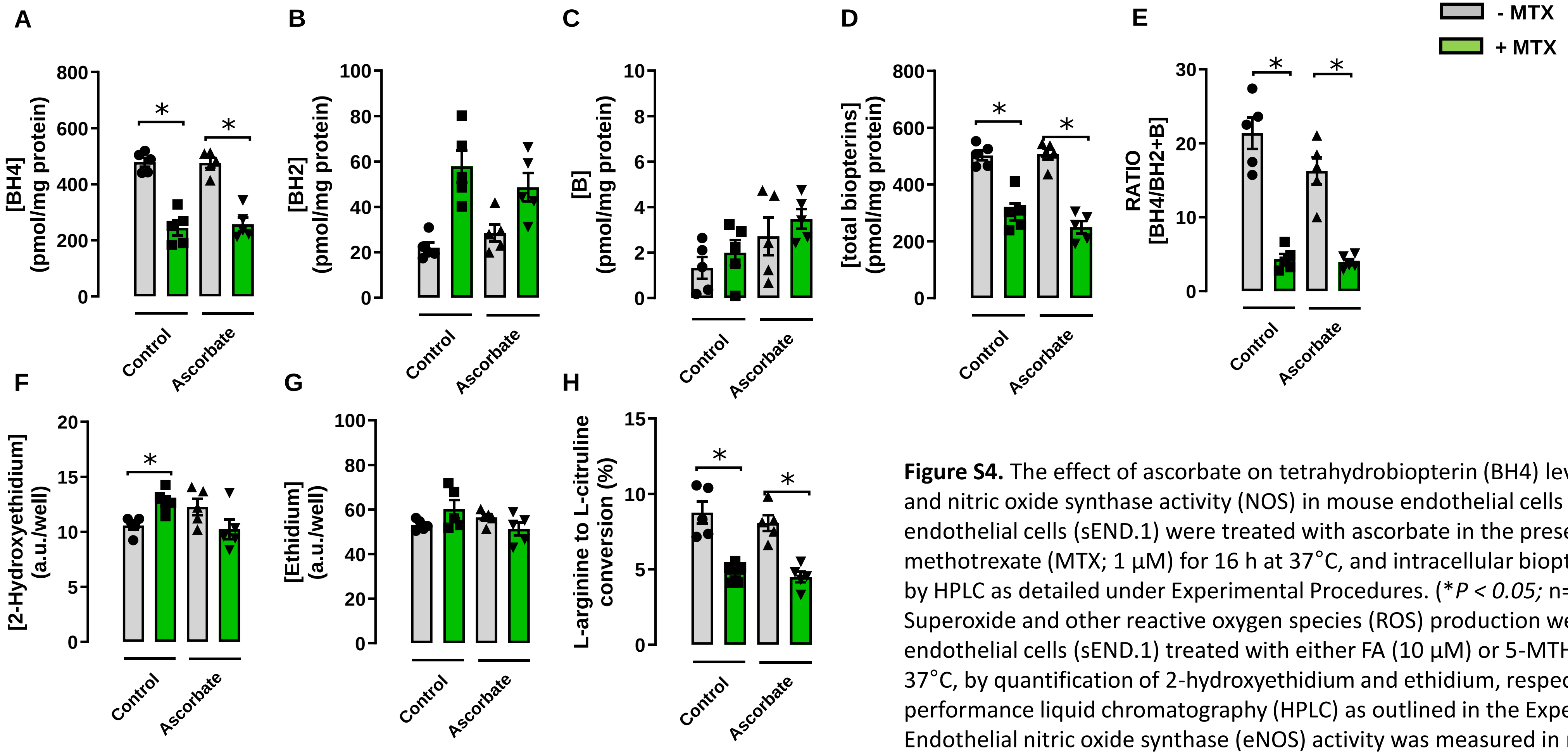

Figure S4. The effect of ascorbate on tetrahydrobiopterin (BH4) levels, superoxide production and nitric oxide synthase activity (NOS) in mouse endothelial cells (sEND1). (**A-E**) Mouse endothelial cells (sEND.1) were treated with ascorbate in the presence or absence of methotrexate (MTX; 1 µM) for 16 h at 37°C, and intracellular biopterin levels were quantified by HPLC as detailed under Experimental Procedures. (*$P < 0.05$; n=5 per group). (**F-G**) Superoxide and other reactive oxygen species (ROS) production were measured in mouse endothelial cells (sEND.1) treated with either FA (10 µM) or 5-MTHF (10 µM) for 16 h at 37°C, by quantification of 2-hydroxyethidium and ethidium, respectively, using high-performance liquid chromatography (HPLC) as outlined in the Experimental procedures. (**H**) Endothelial nitric oxide synthase (eNOS) activity was measured in mouse endothelial cells treated with ascorbate by conversion of ^{14}C L-arginine in endothelial cell culture, followed by radiochemical HPLC quantification of ^{14}C L-citrulline production. (*$P < 0.05$ n=5 per group). Data are shown as means ± S.E.M.

Figure S5

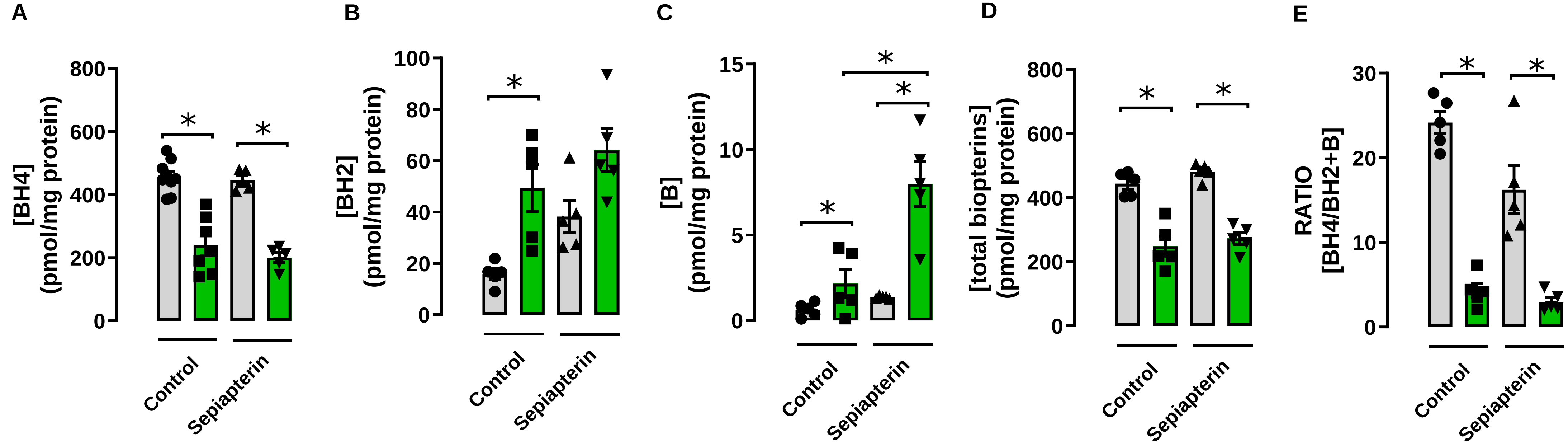

Figure S5. The effect of sepiapterin on tetrahydrobiopterin (BH4) levels in mouse endothelial cells (sEND1). (**A-E**) Mouse endothelial cells (sEND.1) were treated with sepiapterin (1 µM) in the presence or absence of methotrexate (MTX; 1 µM) for 16 h at 37°C, and intracellular biopterin levels were quantified by HPLC as detailed under Experimental Procedures. (**P < 0.05;* n=5 to 9 per group). Data are shown as means ± S.E.M.

Figure S6

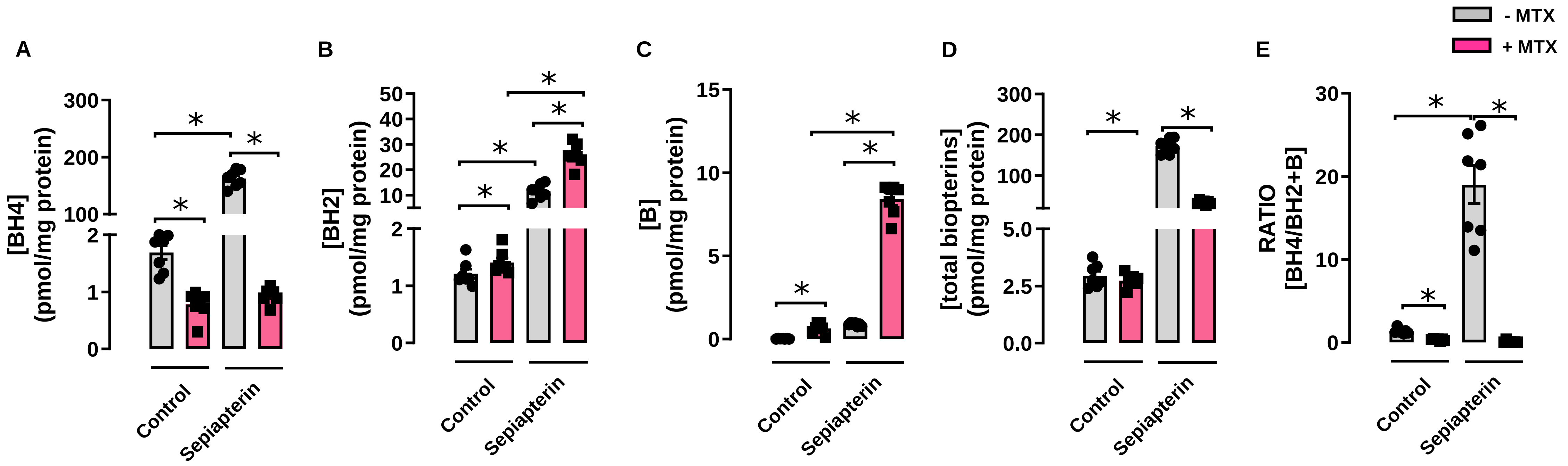

Figure S6. The effect of sepiapterin on tetrahydrobiopterin (BH4) levels in human umbilical vein endothelial cells (HUVECS). (**A-E**) HUVECS were treated with sepiapterin (1 μM) in the presence or absence of methotrexate (MTX; 1 μM) for 16 h at 37°C, and intracellular biopterin levels were quantified by HPLC as detailed under Experimental Procedures. (**P* < 0.05*; n=7 per group). Data are shown as means ± S.E.M.
